# Zoonotic *Cryptosporidium* Species in Animals Inhabiting Sydney Water Catchments

**DOI:** 10.1371/journal.pone.0168169

**Published:** 2016-12-14

**Authors:** Alireza Zahedi, Paul Monis, Sarah Aucote, Brendon King, Andrea Paparini, Fuchun Jian, Rongchang Yang, Charlotte Oskam, Andrew Ball, Ian Robertson, Una Ryan

**Affiliations:** 1 Vector and Waterborne Pathogen Research Group, School of Veterinary and Life Sciences, Murdoch University, Perth, Australia; 2 Australian Water Quality Centre, South Australian Water Corporation, Adelaide, Australia; 3 School of Medicine, Flinders University, Bedford Park, Australia; 4 Henan Agricultural University, Henan, China; 5 WaterNSW, Sydney, Australia; NIH, UNITED STATES

## Abstract

*Cryptosporidium* is one of the most common zoonotic waterborne parasitic diseases worldwide and represents a major public health concern of water utilities in developed nations. As animals in catchments can shed human-infectious *Cryptosporidium* oocysts, determining the potential role of animals in dissemination of zoonotic *Cryptosporidium* to drinking water sources is crucial. In the present study, a total of 952 animal faecal samples from four dominant species (kangaroos, rabbits, cattle and sheep) inhabiting Sydney’s drinking water catchments were screened for the presence of *Cryptosporidium* using a quantitative PCR (qPCR) and positives sequenced at multiple loci. *Cryptosporidium* species were detected in 3.6% (21/576) of kangaroos, 7.0% (10/142) of cattle, 2.3% (3/128) of sheep and 13.2% (14/106) of rabbit samples screened. Sequence analysis of a region of the 18S rRNA locus identified *C*. *macropodum* and *C*. *hominis* in 4 and 17 isolates from kangaroos respectively, *C*. *hominis* and *C*. *parvum* in 6 and 4 isolates respectively each from cattle, *C*. *ubiquitum* in 3 isolates from sheep and *C*. *cuniculus* in 14 isolates from rabbits. All the *Cryptosporidium* species identified were zoonotic species with the exception of *C*. *macropodum*. Subtyping using the 5’ half of *gp60* identified *C*. *hominis* IbA10G2 (n = 12) and IdA15G1 (n = 2) in kangaroo faecal samples; *C*. *hominis* IbA10G2 (n = 4) and *C*. *parvum* IIaA18G3R1 (n = 4) in cattle faecal samples, *C*. *ubiquitum* subtype XIIa (n = 1) in sheep and *C*. *cuniculus* VbA23 (n = 9) in rabbits. Additional analysis of a subset of samples using primers targeting conserved regions of the MIC1 gene and the 3’ end of *gp60* suggests that the *C*. *hominis* detected in these animals represent substantial variants that failed to amplify as expected. The significance of this finding requires further investigation but might be reflective of the ability of this *C*. *hominis* variant to infect animals. The finding of zoonotic *Cryptosporidium* species in these animals may have important implications for the management of drinking water catchments to minimize risk to public health.

## Introduction

*Cryptosporidium* is one of the most prevalent waterborne parasitic infections [1] and represents a public health concern of water utilities in developed countries, including Australia. Currently, 31 *Cryptosporidium* species have been recognised based on biological and molecular characteristics including two recently described species; *C*. *proliferans* and *C*. *avium* [2, 3, 4, 5, 6]. Of these, *C*. *parvum* and *C*. *hominis* have been responsible for all waterborne outbreaks typed to date, with the exception of a single outbreak in the UK caused by *C*. *cuniculus* [7, 8, 9].

In Australia, marsupials, rabbits, sheep and cattle are the dominant animals inhabiting drinking water catchments and can contribute large volumes of manure to water sources [10]. Therefore, it is important to understand the potential contribution from these animals in terms of *Cryptosporidium* oocyst loads into surface water. A number of genotyping studies have been conducted on animals in Australian water catchments to date and have reported a range of species including *C*. *parvum*, *C*. *hominis*, *C*. *cuniculus*, *C*. *ubiquitum*, *C*. *bovis*, *C*. *ryanae*, *C*. *canis*, *C*. *macropodum*, *C*. *fayeri*, *C*. *xiaoi*, *C*. *scrofarum*, and *C*. *andersoni* [11, 12, 13, 14, 15, 16, 17, 18, 19, 20, 21, 22, 23]. To date, in humans in Australia, *C*. *hominis*, *C*. *parvum*, *C*. *meleagridis*, *C*. *fayeri*, *C*. *andersoni*, *C*. *bovis*, *C*. *cuniculus*, a novel *Cryptosporidium* species most closely related to *C*. *wrairi* and the *Cryptosporidium* mink genotype have been reported [24, 25, 26, 27, 28, 29, 30, 31, 32, 33, 34, 35, 36, 37, 38, 39, 40, 41, 42]. The aim of the present study was to use molecular tools to identify the *Cryptosporidium* sp. infecting the kangaroos, rabbits, cattle and sheep population inhabiting Sydney’s drinking water catchments and so better understand the potential health risks they pose.

## Materials and Methods

### Sample collection and processing

Animal faecal samples were collected by WaterNSW staff from watersheds within the WaterNSW area of operations. Sampling was carried out either on land owned by WaterNSW or on private land owned by farmers who gave permission to WaterNSW staff to conduct this study on their property. To minimize cross-contamination and avoid re-sampling the same animals, animals were observed defecating and then samples were collected randomly from freshly deposited faces from the ground, using a scrapper to expose and scoop from the center of the scat pile. Samples were collected on a monthly interval over an 18 months period (July, 2013 to February, 2015) into individual 75 ml faecal collection pots, and stored at 4°C until required (no animal was sacrificed). As faecal samples were collected from the ground and not per rectum, animal ethics approval was not required. Instead, an animal cadaver/tissue notification covering all the samples collected was supplied to the Murdoch University Animal Ethics Committee. The animal sources of the faecal samples were confirmed by watching the host defecate prior to collection and also with the aid of a scat and tracking manual published for Australian animals [43]. Faecal samples were collected from two previously identified hotspot zones from eastern grey kangaroos (*Macropus giganteus*) (n = 576), cattle (n = 142), sheep (n = 128) and rabbits (n = 106). This study did not involve collecting samples from endangered or protected animal species. Samples were shipped to Murdoch University and stored at 4°C until required.

### Enumeration of *Cryptosporidium* oocysts in faecal samples

Enumeration of *Cryptosporidium* oocysts by microscopy was conducted in duplicate for a subset of samples (n = 8) by Australian Laboratory Services (Scoresby, Vic). To quantify recovery efficiency, each individual faecal composite or homogenate was seeded with ColorSeed (Biotechnology Frontiers Ltd. [BTF], Sydney, Australia). *Cryptosporidium* oocysts were purified from faecal samples using immunomagnetic separation (IMS) employing the Dynal GC Combo kit (Dynal, Oslo, Norway) as described by Cox et al., (2005) [44]. Oocysts were stained with Easystain and 4’,6’,-diamidino-2-phenylindole (DAPI; 0.8 μg.ml^-1^) (Biotechnology Frontiers Ltd. [BTF], Sydney, Australia) and examined with an Axioskop epifluorescence microscope (Zeiss, Germany) using filter set 09 (blue light excitation) for Easystain (BTF), filter set 02 (UV light excitation) for DAPI staining, and filter set 15 (green light excitation) for ColorSeed (BTF). The identification criteria described in U.S. EPA method 1623 [45] were used for Easystain-labeled and DAPI-stained objects.

### DNA isolation

Genomic DNA was extracted from 250mg of each faecal sample using a Power Soil DNA Kit (MO BIO, Carlsbad, California). A negative control (no faecal sample) was used in each extraction group.

### PCR amplification of the 18S rRNA gene

All samples were screened for the presence of *Cryptosporidium* at the 18S rRNA locus using a quantitative PCR (qPCR) previously described [46, 47]. qPCR standards were *Cryptosporidium* oocysts (purified and haemocytometer counted), diluted to a concentration of 10,000 oocysts/μl. DNA was extracted from this stock using a Powersoil DNA extraction kit (MO BIO, Carlsbad, California, USA). The 10,000 oocyst/μl DNA stock was then serially diluted to create oocyst DNA concentrations equivalent to 1000, 100, 10, 1 oocysts/μl DNA respectively to be used for standard curve generation using Rotor-Gene 6.0.14 software. Absolute numbers of *Cryptosporidium* oocysts in these standards were determined using droplet digital PCR (ddPCR) at the 18S locus using the same primer set and these ddPCR calibrated standards were used for qPCR as previously described [47]. Each 10 μl PCR mixture contained 1x Go Taq PCR buffer (KAPA Biosystems), 3.75 mM MgCl_2_, 400 μM of each dNTP, 0.5 μM 18SiF primer, 0.5 μM 18SiR primer, 0.2 μM probe and 1U/reaction Kapa DNA polymerase (KAPA Biosystems). The PCR cycling conditions consisted of one pre-melt cycle at 95°C for 6 min and then 50 cycles of 94°C for 20 sec and 60°C for 90 sec.

Samples that were positive by qPCR were amplified at the 18S locus using primers which produced a 611 bp product (Table 1) as previously described [48] with minor modifications; the annealing temperature used in the present study was 57°C for 30 sec and the number of cycles was increased from 39 to 47 cycles for both primary and secondary reactions. PCR contamination controls were used including negative controls and separation of preparation and amplification areas. A spike analysis (addition of 0.5 μL of positive control DNA into each sample) at the 18S locus by qPCR, was conducted on randomly selected negative samples from each group of DNA extractions to determine if negative results were due to PCR inhibition, by comparing the Ct of the spike and the positive control (both with same amount of DNA).

**Table 1:** List of primers used in this study to amplify *Cryptosporidium* species at 18S, lectin (*Clec)*, *gp60*, *lib13* and *MIC1* gene loci.

Gene	Forward Primer	Reverse Primer	Reference
**18S**	5′ ACCTATCAGCTTTAGACGGTAGGGTAT 3′	5′ TTCTCATAAGGTGCTGAAGGAGTAAGG 3′	[48]
	5′ ACAGGGAGGTAGTGA CAAGAAATAACA 3′	5′ AAGGAGTAAGGAACAACCTCCA 3′	
**lectin (*Clec*)**	5′ TCAACTAACGAAGGAGGGGA 3’	5′ GTGGTGTAGAATCGTGGCCT 3′	Present Study
	5′ CCAACATACCATCCTTTGG 3′	5′ GTGGTGTAGAATCGTGGCCT 3′	
***gp60***	5′ ATAGTCTCGCTGTATTC 3′	5′ GCAGAGGAACCAGCATC 3′	[49, 50]
	5′ TCCGCTGTATTCTCAGCC 3′	5′ GAGATATATCTTGGTGCG 3′	
**18S**	5′ TTCTAGAGCTAATACATGCG 3′	5′ CCCATTTCCTTCGAAACAGGA 3′	[51, 52]
	5′ CCCATTTCCTTCGAAACAGGA 3′	5′ CTCATAAGGTGCTGAAGGAGTA 3′	
***gp*60**	5′ ATAGTCTCCGCTGTATTC 3′	5′ GGAAGGAACGATGTATCT 3′	[52, 53]
	5′ GGAAGGGTTGTATTTATTAGATAAAG 3′	5′ GCAGAG GAA CCAGCAT 3′	
***lib13***	5′ TCCTTGAAATGAATATTTGTGACTCG 3′	5′ AAATGTGGTAGTTGCGGTTGAAA 3′	[54]
	Probe: VIC-CTTACTTCGTGGCGGCGT MGB-NFQ		
***MIC1***	5′ TGCAGCACAAACAGTAGATGTG 3′	5′ ATAAGGATCTGCCAAAGGAACA 3′	[52]
	5′ ACCGGAATTGATGAGAAATCTG 3′	5′ CATTGAAAGGTTGACCTGGAT 3′	

### PCR amplification of the lectin (*Clec*) gene

Samples that were typed as *C*. *parvum*, *C*. *hominis* and *C*. *cuniculus* at the 18S locus were also typed using sequence analysis at a unique *Cryptosporidium* specific gene (*Clec*) that codes for a novel mucin-like glycoprotein that contains a C-type lectin domain [55, 56]. Hemi-nested primers were designed for this study using MacVector 12.6 (http://www.macvector.com). The external primers Lectin F1 5’ TCAACTAACGAAGGAGGGGA 3’ and Lectin R1 5’ GTGGTGTAGAATCGTGGCCT 3’ produced a fragment size of 668 bp for *C*. *hominis* and 656 bp for *C*. *parvum*. The secondary reaction consisted of primers, Lectin F2 5’ CCAACATACCATCCTTTGG 3’ and Lectin R1 5’ GTGGTGTAGAATCGTGGCCT 3’ (Table 1), which produced a fragment of 518 bp for *C*. *hominis*, 506 bp for *C*. *parvum* and 498 bp for *C*. *cuniculus*. The cycling conditions for the primary amplification was 94°C for 3 min, followed by 94°C for 30 sec, 58°C for 30 sec, 72°C for 1 min for 40 cycles, plus 5 min at 72°C for the final extension. The same cycling conditions were used for the secondary PCR, with the exception that the number of cycles was increased to 47 cycles. The 25 μl PCR mixture consisted of 1 μl of DNA, 1x Go Taq PCR buffer (KAPA Biosystems), 200 μM of each dNTP (Promega, Australia), 2 mM MgCl_2_, 0.4 μM of each primer, 0.5 units of Kapa DNA polymerase (KAPA Biosystems). The specificity of this locus for *Cryptosporidium* has been previously confirmed [41]. Enumeration of *Cryptosporidium* oocysts by qPCR was conducted using a specific *C*. *hominis* and *C*. *parvum* assay targeting the *Clec* gene as previously described [41].

### PCR amplification of the *gp60* gene

Samples that were typed as *C*. *hominis*, *C*. *parvum*, *C*. *cuniculus* and *C*. *ubiquitum* at the 18S locus were subtyped at the 60 kDa glycoprotein (*gp60*) locus using nested PCR as previously described (Table 1) [57, 49 50, 58].

### Sequence analysis and phylogenetic analysis

The amplified DNA from secondary PCR products were separated by gel electrophoresis and purified for sequencing using an in house filter tip method [41]. Purified PCR products from all three loci, were sequenced independently using an ABI Prism^™^ Dye Terminator Cycle Sequencing kit (Applied Biosystems, Foster City, California) according to the manufacturer’s instructions at 57°C, 58°C and 54°C annealing temperature for the 18S rRNA, lectin and *gp60* loci, respectively. Sanger sequencing chromatogram files were imported into Geneious Pro 8.1.6 [59], edited, analysed and aligned with reference sequences from GenBank using ClustalW (http://www.clustalw.genome.jp). Distance, parsimony and maximum likelihood trees were constructed using MEGA version 7 [60].

### Independent confirmation by the Australian Water Quality Centre (AWQC)

A total of eight blinded faecal samples consisting of seven *C*. *hominis* positives and one *Cryptosporidium* negative were sent to the Australian Water Quality Centre (AWQC) for independent analysis. DNA was extracted using a QIAamp DNA Mini extraction kit (Qiagen, Australia). Samples were screened using primers targeting the 18S rRNA locus (Xiao et al., 2000 as modified by Webber at al., 2014) [51, 52], *gp60* using producing an approx. 871 bp secondary product (Alves et al., 2003 as modified by Webber at al., 2014) [53, 52] and an approx. 400 bp primary product [50] as well as the *lib13* [54] and *MIC1* gene loci [52] as previously described (Table 1). PCRs were conducted on a RotorGene 6000 HRM (Qiagen) or LightCycler 96 (Roche) and amplification of the correct product was determined by DNA melting curve analysis [52]. Amplicons with atypical DNA melting profiles were further characterized by capillary electrophoresis using a DNA 1000 chip on a Bioanalyzer 2100 (Agilent) as per the manufacturer’s instructions. The amplicons from all positive PCRs were purified using a Qiagen PCR purification kit according to the manufacturer's instructions and submitted to the Australian Genome Research Facility for DNA sequencing using BigDye3 chemistry on an Applied Biosystems AB3730xl capillary DNA sequencer. Sequences were analyzed using Geneious Pro 6.1.8 (Biomatters).

### PCR amplification of open reading frames flanking *gp60* and *MIC1*

Open reading frames flanking both ends of *gp60* and *MIC1* in the *C*. *parvum* genome were used in BLAST searches (http://blast.ncbi.nlm.nih.gov/) to obtain homologous *C*. *hominis* sequences. Alignments of the *C*. *parvum* and *C*. *hominis* open reading frame pairs were constructed using Geneious Pro 6.1.8 (Biomatters). Conserved primers were designed for each alignment using the default settings and a target amplicon size of approximately 400 bp. The resulting primers (Table 2) were subjected to BLAST searches to verify specificity.

**Table 2:** List of primers designed in the present study to amplify regions flanking the 5’ and 3’ ends of *MIC1* and *gp60*.

Gene	Flanking openreading frame	Forward Primer	Reverse Primer	Product size (*C*. *parvum* and *C*. *hominis*)
***MIC1***	cgd6_770 Chro. 60100(3’ end)hypothetical proteinCDS	5’TGCGGTTGTATGACACCATCA 3’	5’TCTCTGGTGTTTGGCCTGAC 3’	511
	cgd6_810 Chro. 60105(5’ end)BRCT	5’AGACACCAAGATGGAAAAGGCA 3’	5’GGGAAGACCTTTTGATATTGCCC 3’	467
***gp60***	cgd6_1070 Chro. 60137(3’ end)conservedhypothetical protein	5’AGCAAGACCGCAACTCAAGT 3’	5’CCCATAGTGCCCAGCTTGAA 3’	430
	cgd6_1090 Chro. 60141(5’ end) hsp40	5’TATTTGGAGGTGGGGCCAAG 3’	5’AAAACGGGTTTAGGGGTGGT 3’	367

Each 25 μl qPCR reaction contained 0.5 x GoTaq PCR Buffer (Promega), 1.5 mM MgCl_2_, 0.2 mM dNTP, 3.3 μM SYTO 9, 100 ng GP32, 0.5 μM forward primer, 0.5 μM reverse primer, 1 unit Promega GoTaq HS, and 2 μl of DNA extract. The qPCR was performed on a Light Cycler96 (Roche), and cycling conditions consisted of one pre-melt cycle at 95°C for 6 min and then 40 cycles of 94°C for 45 sec, 60°C for 45 sec and 72°C for 60 sec. High-resolution DNA melting curve analysis was conducted from 65°C to 97°C using an acquisition rate of 25 reads /°C. *Blastocystis hominis* DNA was used as a negative control and nuclease free water was used as a no template control. Positive controls included *C*. *parvum* Iowa 2a (BTF, Sydney, Australia) and *C*. *hominis* IbA10G2 (kindly provided by Ika Sari). Amplicons were sized by capillary electrophoresis using a DNA 1000 chip on a Bioanalyzer 2100 (Agilent) as per the manufacturer’s instructions.

### Statistical Analysis

The prevalence of *Cryptosporidium* in faecal samples collected from each host species was expressed as the percentage of samples positive by qPCR, with 95% confidence intervals calculated assuming a binomial distribution, using the software Quantitative Parasitology 3.0 [61]. Linear coefficients of determination (R^2^) and Spearman's rank correlation coefficient (Spearman's rho) were used for the analysis of agreement (correlation) between oocyst numbers per gram of faeces determined by qPCR calibrated with ddPCR standards and enumeration of *Cryptosporidium* oocysts by microscopy (IMS) using SPSS 21.0 for Windows (SPSS Inc. Chicago, USA).

## Results

### Prevalence of *Cryptosporidium* in faecal samples collected from various hosts

The overall PCR prevalence of *Cryptosporidium* species in 952 faecal samples collected from four different host species was 5% (48/952) (Table 3). *Cryptosporidium* species were detected in 3.6% (21/576) of the kangaroo faecal samples, 7.0% (10/142) of cattle faeces, 2.3% (3/128) of sheep faeces and 13.2% (14/106) of rabbit faecal samples based on qPCR and sequence analysis of the 18S rRNA locus (Table 3).

**Table 3:** Prevalence of *Cryptosporidium* species in faecal samples collected from four different host species in Sydney water catchments*. 95% confidence intervals are given in parenthesis.

Host species	Number of samples	Number of positives	Prevalence%	Species and subtype
**Eastern grey kangaroo**	576	21	3.6 (95% CI: 2.3–5.5)	*C*. *hominis* (n = 17)**,IbA10G2 (n = 12),IdA15G1 (n = 2),*C*. *macropodum* (n = 4)
**Cattle**	142	10	7 (95% CI: 3.4–12.6)	*C*. *hominis* (n = 6)**,IbA10G2 (n = 4),*C*. *parvum* (n = 4),IIaA18G3R1 (n = 4)
**Sheep**	128	3	2.3 (95% CI: 0.5–6.7)	*C*. *ubiquitum* (n = 3)**,XIIa (1)
**Rabbit**	106	14	13.2 (95% CI: 7.4–21.2)	*C*. *cuniculus* (n = 14)**,VbA 23 (n = 9)
**Total**	**952**	**48**	**5 (95% CI: 3.7–6.6)**	

* Based on PCR amplification and sequencing at the 18S rRNA gene, with subtyping based on DNA sequence analysis of a 400 bp amplicon from the 5’ end of the *gp60* locus.

** Not all positive samples were successfully typed.

### *Cryptosporidium* species detected in various hosts

Sequencing of secondary PCR amplicons at the 18S rRNA locus identified four of the 21 positive isolates from kangaroo faecal samples as *C*. *macropodum*, while the other 17 isolates were identified as *C*. *hominis* (100% similarity for 550bp) (Table 4). Of the ten positives detected in cattle faecal samples, six were *C*. *hominis* and four were *C*. *parvum* (Table 4). The three sheep positive samples were identified as *C*. *ubiquitum* and all fourteen positives detected in rabbit faecal samples were *C*. *cuniculus* (Table 4).

**Table 4:** Species and subtypes of *Cryptosporidium* identified in faecal samples from various hosts (and their GPS co-ordinates) at the 18S and *gp60* loci.

Host species	Southing	Easting	18S locus	*gp*60 locus
**Eastern grey kangaroo 1**	-34.18861	150.2918	*C*. *hominis*	*C*. *hominis* IbA10G2
**Eastern grey kangaroo 2**	-34.203794	150.284394	*C*. *macropodum*	-
**Eastern grey kangaroo 3**	-34.20207	150.2742	*C*. *hominis*	*C*. *hominis* IbA10G2
**Eastern grey kangaroo 4**	-34.193631	150.273387	*C*. *macropodum*	-
**Eastern grey kangaroo 5**	-34.188607	150.291818	*C*. *macropodum*	-
**Eastern grey kangaroo 6**	-34.20458	150.2881	*C*. *hominis*	*C*. *hominis* IbA10G2
**Eastern grey kangaroo 7**	-34.61547	150.59756	*C*. *hominis*	no amplification
**Eastern grey kangaroo 8**	-34.23796	150.2598	*C*. *hominis*	*C*. *hominis* IbA10G2
**Eastern grey kangaroo 9**	N/A	N/A	*C*. *hominis*	*C*. *hominis* IbA10G2
**Eastern grey kangaroo 10**	N/A	N/A	*C*. *hominis*	*C*. *hominis* IbA10G2
**Eastern grey kangaroo 11**	N/A	N/A	*C*. *hominis*	*C*. *hominis* IbA10G2
**Eastern grey kangaroo 12**	N/A	N/A	*C*. *hominis*	*C*. *hominis* IbA10G2
**Eastern grey kangaroo 13**	-34.61686	150.68794	*C*. *hominis*	*C*. *hominis* IbA10G2
**Eastern grey kangaroo 14**	-34.63269	150.619	*C*. *hominis*	*C*. *hominis* IbA10G2
**Eastern grey kangaroo 15**	-34.63269	150.61897	*C*. *hominis*	no amplification
**Eastern grey kangaroo 16**	-34.61422	150.59331	*C*. *hominis*	*C*. *hominis* IbA15G1
**Eastern grey kangaroo 17**	-34.61415	150.59376	*C*. *hominis*	*C*. *hominis* IbA10G2
**Eastern grey kangaroo 18**	-34.61686	150.68794	*C*. *hominis*	no amplification
**Eastern grey kangaroo 19**	-31.60846	150.60819	*C*. *macropodum*	-
**Eastern grey kangaroo 20**	-34.61472	150.68475	*C*. *hominis*	*C*. *hominis* IbA10G2
**Eastern grey kangaroo 21**	-34.61472	150.68475	*C*. *hominis*	*C*. *hominis* IbA15G1
**Cattle 1**	-34.61278	150.585	*C*. *hominis*	no amplification
**Cattle 2**	-34.60429	150.60170	*C*. *hominis*	*C*. *hominis* IbA10G2
**Cattle 3**	-34.61283	150.58514	*C*. *hominis*	no amplification
**Cattle 4**	-34.60429	150.60170	*C*. *parvum*	*C*. *parvum* IIaA18G3R1
**Cattle 5**	-34.60642	150.60126	*C*. *parvum*	*C*. *parvum* IIaA18G3R1
**Cattle 6**	-34.61373	150.5876	*C*. *parvum*	*C*. *parvum* IIaA18G3R1
**Cattle 7**	-34.61373	150.5876	*C*. *hominis*	*C*. *hominis* IbA10G2
**Cattle 8**	-34.6195	150.5242	*C*. *hominis*	*C*. *hominis* IbA10G2
**Cattle 9**	-34.60429	150.60170	*C*. *hominis*	*C*. *hominis* IbA10G2
**Cattle 10**	-34.63269	150.619	*C*. *parvum*	*C*. *parvum* IIaA18G3R1
**Sheep 1**	-34.61556	150.68353	*C*. *ubiquitum*	no amplification
**Sheep 2**	-34.61556	150.68353	*C*. *ubiquitum*	no amplification
**Sheep 3**	-34.61743	150.68674	*C*. *ubiquitum*	*C*. *ubiquitum* XIIa
**Rabbit 1**	-34.61954	150.62169	*C*. *cuniculus*	no amplification
**Rabbit 2**	-34.61959	150.62172	*C*. *cuniculus*	*C*. *cuniculus* VbA23
**Rabbit 3**	-34.61937	150.62178	*C*. *cuniculus*	*C*. *cuniculus* VbA23
**Rabbit 4**	-34.61479	150.68492	*C*. *cuniculus*	*C*. *cuniculus* VbA23
**Rabbit 5**	-34.61954	150.62169	*C*. *cuniculus*	no amplification
**Rabbit 6**	-34.6195	150.52415	*C*. *cuniculus*	no amplification
**Rabbit 7**	-34.61937	150.62178	*C*. *cuniculus*	*C*. *cuniculus* VbA23
**Rabbit 8**	-34.61283	150.58514	*C*. *cuniculus*	*C*. *cuniculus* VbA23
**Rabbit 9**	-34.61556	150.68353	*C*. *cuniculus*	*C*. *cuniculus* VbA23
**Rabbit 10**	-34.61278	150.585	*C*. *cuniculus*	no amplification
**Rabbit 11**	-34.61479	150.68492	*C*. *cuniculus*	*C*. *cuniculus* VbA23
**Rabbit 12**	-34.60429	150.60170	*C*. *cuniculus*	*C*. *cuniculus* VbA23
**Rabbit 13**	-34.18951	150.2885	*C*. *cuniculus*	no amplification
**Rabbit 14**	-34.6327	150.619	*C*. *cuniculus*	*C*. *cuniculus* VbA23

Sequence analysis at the lectin *(Clec)* locus was consistent with 18S gene results. Eleven of 17 *C*. *hominis* isolates from kangaroos were successfully amplified and confirmed as *C*. *hominis* sequences. Eight of the 14 positives from rabbits successfully amplified at this locus and were identified as *C*. *cuniculus*. Four of six *C*. *hominis* and all four *C*. *parvum* isolates from cattle were also confirmed at this locus.

Sequences at the *gp60* locus were obtained for 14 kangaroo and four cattle isolates that were typed as *C*. *hominis* at the 18S rRNA locus. These samples failed to amplify at *gp60* using the primers of Strong et al., (2000) or Alves et al., (2003) [57, 53], which amplify an approx. 832 bp fragment, but were successfully amplified using the nested primers by Zhou et al., (2003) [53], which amplify a 400 bp product. In approx. 50% of samples, the primary reaction did not produce a visible band by gel electrophoresis but a band of the correct size was visible for the secondary PCR, which was then confirmed by sequencing.

**Fig 1:**
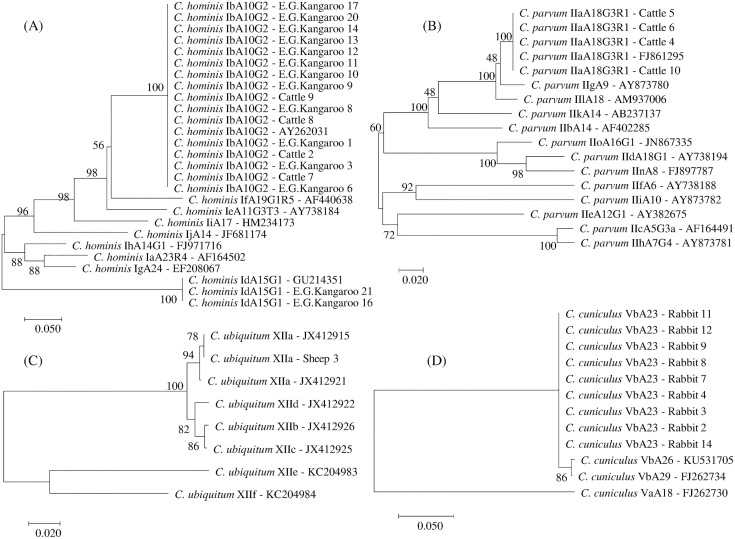
Phylogenetic relationships of *Cryptosporidium* subtypes inferred from Neighbor-Joining (NJ) analysis of Kimura’s distances calculated from pair-wise comparisons of *gp60* sequences. (A) Relationships among *C*. *homi*nis subtypes. (B) Relationships among *C*. *parvum* subtypes. (C) Relationships between *C*. *ubiquitum* subtypes. (D) Relationships between *C*. *cuniculus* subtypes. Percentage support (>50%) from 1000 pseudoreplicates from NJ analyses is indicated at the left of the supported node.

The *C*. *hominis* subtypes IbA10G2 and IdA15G1 were identified in 12 and 2 kangaroo samples respectively and the IbA10G2 subtype was also identified in four cattle samples (Table 4 and Fig 1A). The four *C*. *parvum* isolates from cattle were identified as subtype IIaA18G3R1 and the *C*. *cuniculus* isolates were subtyped as VbA23 (n = 9) (Table 4 and Fig 1B and 1D). Of the three *C*. *ubiquitum* positive isolates at 18S locus, only one isolate was successfully subtyped and identified as *C*. *ubiquitum* subtype XIIa (Table 4 and Fig 1C). Nucleotide sequences reported in this paper are available in the GenBank database under accession numbers; KX375346, KX375347, KX375348, KX375349, KX375350, KX375351, KX375352, KX375353, KX375354, KX375355.

### Independent confirmation by the Australian Water Quality Centre (AWQC)

Blind independent analysis conducted by AWQC using the 18S rRNA nested PCR of Xiao et al., (2000) [51] identified *C*. *hominis* in six samples, corresponding with the six positive samples from kangaroos, and failed to detect *Cryptosporidium* in the other two samples, one of which corresponded with the negative sample. Amplification of a region of *gp60* using the protocol described by Alves et al. [53] failed to produce an amplicon for either the primary or secondary reactions. Amplification of *gp60* using the protocol described by Zhou et al., (2003) [50], failed to amplify the correct-sized product for the primary PCR but produced amplicons of the correct size for the secondary PCR for the six positive samples, which when sequenced were confirmed as *C*. *hominis* subtype IbA10G2. Amplification at the *lib13* locus was also successful for the six positive samples, which were confirmed as *C*. *hominis*. Amplification at the *MIC1* locus failed to produce any amplicons. The *gp60* and *MIC1* amplification failures were further investigated using PCR assays designed to target open reading frames (ORFs) flanking these two loci. All four primer sets produced strong amplification of the correctly sized fragments for the *C*. *parvum* and *C*. *hominis* control DNA. The cgd6-1070 ORF (located downstream of *gp60* in *C*. *parvum*), and cgd6-810 (upstream of *MIC1*), both amplified from four of the six samples identified as *C*. *hominis*. In the case of the other 2 ORFs, weak amplification was observed for one sample for cgd6-1090 (upstream of *gp60*) and for two samples for cgd6-770 (downstream of *MIC1*). While only single bands were observed for the *C*. *parvum* and *C*. *hominis* controls, most of the faecal sample extracts produced multiple bands.

### Enumeration of *Cryptosporidium* oocysts in faecal samples

Oocyst numbers per gram of faeces for all PCR positive samples were determined using qPCR at the *Clec* locus for 18 *C*. *hominis* and 4 *C*. *parvum* positives and for a subset of samples (n = 8) using microscopy (Table 5). For the 8 samples for which both microscopy and qPCR data were available, there was poor correlation between the two methods (*R*^2^ ≈ 0.0095 and ρ (rho) = 0.2026) (Table 5). Based on qPCR, the highest numbers of oocysts was detected in Eastern grey kangaroo isolate 12 (16,890 oocysts/g^-1^), which was identified as *C*. *hominis* subtype IbA10G2. No oocysts (<2g^-1^) were detected by microscopy in this sample.

**Table 5:** *Cryptosporidium* oocyst numbers in positive samples per gram of faeces (g^-1^) determined using microscopy and qPCR. Note: microscopy data was only available for 12 samples.

Host species	*Cryptosporidium* species (18S)	Oocyst numbers/g^-1^ microscopy	% Oocyst recovery	Oocyst numbers/g^-1^ qPCR
**Eastern grey kangaroo 1**	*C*. *hominis*	210	54	11,337
**Eastern grey kangaroo 3**	*C*. *hominis*	11,076	78	5,458
**Eastern grey kangaroo 6**	*C*. *hominis*	<2	61	9,528
**Eastern grey kangaroo 8**	*C*. *hominis*	<2	45	262
**Eastern grey kangaroo 9**	*C*. *hominis*	<2	74	648
**Eastern grey kangaroo 10**	*C*. *hominis*	<2	51	8,735
**Eastern grey kangaroo 11**	*C*. *hominis*	<2	67	131
**Eastern grey kangaroo 12**	*C*. *hominis*	<2	60	16,890
**Eastern grey kangaroo 13**	*C*. *hominis*	-	-	26
**Eastern grey kangaroo 14**	*C*. *hominis*	-	-	5,458
**Eastern grey kangaroo 16**	*C*. *hominis*	-	-	7,570
**Eastern grey kangaroo 17**	*C*. *hominis*	-	-	9,626
**Eastern grey kangaroo 20**	*C*. *hominis*	-	-	8,735
**Eastern grey kangaroo 21**	*C*. *hominis*	-	-	173
**Cattle 2**	*C*. *hominis*	-	-	144
**Cattle 4**	*C*. *parvum*	-	-	936
**Cattle 5**	*C*. *parvum*	-	-	1,819
**Cattle 6**	*C*. *parvum*	-	-	2,197
**Cattle 7**	*C*. *hominis*	-	-	4,205
**Cattle 8**	*C*. *hominis*	-	-	10,827
**Cattle 9**	*C*. *hominis*	-	-	15,804
**Cattle 10**	*C*. *parvum*	-	-	1,190

## Discussion

The present study described the prevalence and molecular characterization of *Cryptosporidium* species in faecal samples collected from kangaroo, cattle, sheep and rabbit faecal samples from Sydney’s drinking water catchments. The overall prevalence of *Cryptosporidium* species in the faecal samples collected from four animal hosts was 5% and was 3.6% in kangaroos, 7% in cattle, 2.3% in sheep and 13.2% in rabbits. Overall, the prevalence of infection with *Cryptosporidium* was generally lower than that reported previously in Sydney catchments; 25.8% [44] 6.7% [62] and 8.5% [16] and Western Australian catchments; 6.7% [13]. In the study by Ng et al., (2011b) [16], the prevalence in eastern grey kangaroos was much higher (16.9%−27/160) than the 3.6% prevalence in kangaroo faecal samples in the present study. The overall prevalence of *Cryptosporidium* species in faecal samples collected from different species in the present study was similar to the 2.8% (56/2,009) prevalence identified in faecal samples from animals in Melbourne water catchments [20]. The lower prevalence in the present study and the Melbourne study may be a consequence of testing a greater numbers of samples, seasonal and/or yearly variation in prevalence and/or proximity to agricultural land.

Based on sequence analysis using the 18S rRNA locus, a total of five *Cryptosporidium* species were identified; *C*. *macropodum* (n = 4), *C*. *hominis* (n = 23), *C*. *parvum* (n = 4), *C*. *ubiquitum* (n = 3) and *C*. *cuniculus* (n = 14). The prospect of livestock and wildlife being reservoirs for *C*. *hominis* has human-health implications, so to verify this finding, a subset of faecal samples was subjected to blinded independent analysis. This additional testing initially identified *C*. *hominis* following sequence analysis of a large fragment of the 18S rRNA gene amplified using the Xiao et al., (2000) [51] nested PCR. It is noteworthy that the Xiao outer 18S PCR produced a clear amplification signal (threshold cycles between 24 and 29 for positive samples), suggesting the presence of reasonable numbers of oocysts with no evidence of PCR inhibition for this relatively large amplicon (approx. 1.2 kilobases). The *lib13* Taqman assay also identified *C*. *hominis* in these same samples. However, amplification of *gp60* using the Alves et al., (2003) [53] nested PCR failed to amplify any *Cryptosporidium*, either as a nested PCR or by direct amplification using the inner primer set. Application of the Zhou et al., (2003) [53] outer *gp60* primers (which are equivalent to the pairing of the Alves outer forward and inner reverse primers) also appeared to be unsuccessful (only four samples produced a band close to the expected size), but the Zhou *gp60* inner PCR amplified the correctly sized amplicon, which was confirmed to be *C*. *hominis* IbA10G2.

The failure to amplify *gp60* using the Alves et al., (2003) and Strong et al., (2000) [57, 53] assays was unexpected, especially considering the high degree of conservation for the primer binding sites across the *C*. *parvum* and *C*. *hominis gp60* subtypes and the successful amplification of the large 18S rRNA gene fragment, which demonstrates that the DNA quantity and quality was sufficient for amplification within the first round of PCR. The lack of amplification at other loci is unlikely to be due to PCR inhibition, as spike analysis indicated no inhibition. To investigate this further, a published PCR assay targeting the MIC1 locus from both *C*. *parvum* and *C*. *hominis* [52] was also tested and failed to amplify the expected fragment from these samples. The MIC1 gene encodes a thrombospondin-like domain-containing protein, which is secreted in sporozoites prior to host cell attachment and localized to the apical complex after microneme discharge [63]. As secreted proteins often play a critical role in determining virulence and host specificity in host-pathogen relationships, it has been hypothesized that MIC1 may play a role in the differences in host range observed between *C*. *parvum* and *C*. *hominis* [52]. Previous analysis of the CryptoDB has identified that both the *gp60* and *MIC1* loci are on chromosome 6 and in close proximity (≈60 kb) [52], and it has previously been reported that these two genes are genetically linked [64]. Given that 3 different *gp60* reverse primers appear to have failed, as well as failure of at least one of the MIC1 primers, it would require the occurrence of multiple individual single nucleotide polymorphisms for the results to be accounted for by point mutations. Alternatively, a truncation or rearrangement on chromosome 6 affecting the 3’ end of *gp60* and *MIC1* could affect these PCR assays. To test for any deletions affecting these loci, PCR assays were developed targeting flanking ORFs. The PCR assays targeting two ORFs in the region between *MIC1* and *gp60* (based on the *C*. *parvum* chromosome 6 map) were positive for some of the samples tested, suggesting that a wholesale deletion is not the cause for the failure to amplify *MIC1* or the entire *gp60*. The other two PCR assays produced equivocal results in the samples, although they yielded strong amplification in the positive controls. The variable sample results may have been due to a combination of the low amount of *Cryptosporidium* DNA present and non-specific amplification from other DNA in the sample extracts. The latter is likely, considering that the positive controls produced a single amplicon, whereas most of the sample extracts yielded multiple fragments of different sizes.

Sequencing of chromosome 6 or the entire genome of this variant *C*. *hominis* is required to determine the underlying cause for the failure to amplify *MIC1* or the larger *gp60* region. Considering the role of *gp60* in host cell adhesion and the hypothesized role of MIC1 in infection, it is possible that changes or loss of key genes involved in host specificity could explain the success of this particular variant of *C*. *hominis* in infecting hosts other than humans. If the function of these genes has been altered to better support infection in non-human hosts, then the infectivity of this variant in humans needs to be re-evaluated.

Of the detected species, all but *C*. *macropodum* have been reported to cause infection in humans at varying frequencies [7, 10]. *Cryptosporidium hominis* and *C*. *parvum* are responsible for the majority of human infections worldwide [7, 6]. In the present study, the prevalence of the variant *C*. *hominis* in kangaroo and cattle faecal samples was 2.9% (95% CI: 1.7%-4.7%) and 4.2% (95% CI: 1.6%-9%) respectively, and the prevalence of *C*. *parvum* in cattle faecal samples was 2.8% (95% CI: 0.8%-7.1%). Both of these parasites have been linked to numerous waterborne outbreaks around the world [7, 1] and although this prevalence is relatively low, both these host species represent a risk of waterborne transmission to humans. A number of previous studies have identified *C*. *hominis/C*. *parvum-*like isolates at the 18S rRNA locus in marsupials including bandicoots (*Isoodon obesulus*), brushtail possums (*Trichosurus vulpecula*), eastern grey kangaroos (*Macropus giganteus*) and brush-tailed rock-wallabies (*Petrogale penicillata*) [65, 66, 67]. However, in those studies, despite efforts, the identification of *C*. *hominis/C*. *parvum* could not be confirmed at other loci. This may be due to low numbers of oocysts and the multi-copy nature of the 18S rRNA gene, which provides better sensitivity at this locus. Alternatively, failure to confirm identity in these other studies could be due the presence of variants with substantial differences in the diagnostic loci used, causing those PCR assays to fail. Such is the case in the present study, which for the first time has identified a novel *C*. *hominis* in kangaroo faecal samples based on analysis of multiple loci (18S rRNA, *Clec*, *MIC1*, *lib13* and *gp60*).

*Cryptosporidium cuniculus*, the most prevalent species detected here (13.2%), has been previously identified in rabbits, humans and a kangaroo in Australia [14, 20, Sari et al., 2013 unpublished—KF279538, 21]. It was implicated in a waterborne outbreak of cryptosporidiosis in humans in England in 2008 [8, 9] and has been linked to a number of sporadic human cases across the UK [68, 69], Nigeria [70] and France [71]. *Cryptosporidium ubiquitum* was detected in three sheep samples and is a common human pathogen [7], but has not been identified in Australia in the limited typing of Australian human *Cryptosporidium* isolates that has been conducted to date [10], however it has been identified in surface waters in Australia (Monis et al., unpublished).

Subtyping at the *gp60* locus identified the *C*. *hominis* subtype IbA10G2 in twelve kangaroo and four cattle faecal samples. This is a dominant subtype responsible for *C*. *hominis*-associated outbreaks of cryptosporidiosis in the United States, Europe and Australia [7, 72, 73, 74]. *Cryptosporidium hominis* has previously been reported in cattle in New Zealand [75], Scotland [76], India [77] and Korea [78]. Subtyping at the *gp60* locus identified IbA10G2 [76, 75], and IdA15G1 [77]. It has been suggested that the IbA10G2 infects cattle naturally in particular circumstances and thus could act as a zoonotic infection source in some instances [76]. Interestingly, the studies that detected IbA10G2 in cattle, used PCR-based assays that only sequenced the 5’ end of *gp60*, similar to the assay used in this study, so it is possible that these reports also represent detection of a variant *C*. *hominis gp60*. This is the first report of the same subtype of *C*. *hominis* in kangaroos and cattle in the same catchment. In two kangaroo samples, the *C*. *hominis* IdA15G1 subtype was identified. This is also a common *C*. *hominis* subtype identified in humans worldwide [28, 79, 80, 81, 74]. The source and human health significance of the novel *C*. *hominis* detected in kangaroo and cattle samples in the present study is currently unknown. Environmental pollution from human and domestic animal faeces such as contamination of watersheds due to anthropogenic and agricultural activities conducted in the catchment area, in particular livestock farming, could be a potential source for wildlife infections with *C*. *hominis*. However, further studies are required to better understand the involvement of humans and livestock in the epidemiology of zoonotic *Cryptosporidium* species in wildlife.

The *C*. *parvum* subtype IIaA18G3R1 was identified in four cattle samples. IIaA18G3R1 is also a common subtype in both humans and cattle worldwide and has been reported widely in both calves and humans in Australia [10]. Subtyping of the single *C*. *ubiquitum* isolate from sheep identified XIIa. To date six subtype families (XIIa to XIIf) have been identified in *C*. *ubiquitum* [58]. Of these, XIIa, XIIb, XIIc, and XIId have been found in humans and therefore XIIa is a potentially zoonotic subtype [54] The *C*. *cuniculus* subtype identified in the present study was VbA23. Two distinct *gp60* subtype families, designated Va and Vb have been identified in *C*. *cuniculus* [8]. Most cases described in humans relate to clade Va and the first waterborne outbreak was typed as VaA22 [82, 8]. Previous studies in Australia have identified subtype VbA26 from an Eastern grey kangaroo [42], subtypes VbA23R3 and VbA26R4 [14, 20], VbA22R4, VbA24R3 and VbA25R4 [20] in rabbits and subtype VbA25 [42] and VbA27 (Sari et al., 2013 unpublished—KF279538) in a human patient.

Accurate quantification of *Cryptosporidium* oocysts in animal faecal deposits on land is important for estimating catchment *Cryptosporidium* loads. In the present study, oocyst concentration (numbers per gram of faeces—g^-1^) was also determined for 18 *C*. *hominis* and 4 *C*. *parvum* positives using qPCR and for a subset of samples (n = 8) by microscopy. qPCR quantitation was conducted at the *Clec* locus rather than the 18S rRNA locus as the former is unique to *Cryptosporidium* and therefore more specific than the available 18S rRNA qPCR assays. There was poor correlation between qPCR and microscopy for the 8 samples for which data from both methods were available, with qPCR detecting higher numbers of oocysts than microscopy with the exception of one sample (Eastern grey kangaroo 3). Increased sensitivity of qPCR and the estimation of much higher numbers of oocysts in faecal samples by qPCR versus microscopy has been previously reported [83]. A major limitation of qPCR is that the quantitative data generated are only as accurate as the standards used. A study which compared droplet digital PCR (ddPCR) (which provides absolute quantitation without the need for calibration curves) with qPCR, reported that qPCR overestimated the oocysts counts compared to ddPCR [47]. In the present study, the discrepancy between qPCR and microscopy could be due to a number of different factors; (1) IMS for microscopy and direct DNA extraction from faeces were conducted on different subsamples of each faecal sample and therefore the numbers of oocysts present in the subsamples may differ, (2) microscopy counts intact oocysts whereas qPCR will detect not only oocysts but also sporozoites that have been released from oocysts, other lifecycle stages and any free DNA, therefore qPCR may produce higher counts than microscopy. In the present study, the mean oocysts g^-1^ for kangaroos and cattle that were positive for *C*. *hominis* was 6,041 (range 26–16,890) and for cattle that were positive for *C*. *parvum* was 1535(range 936–2,197) as determined by PCR. By microscopy, oocysts counts were available for kangaroo samples only and the mean was 5,643 (range <0.5–11,076). A previous study in WaterNSW catchments, reported mean *Cryptosporidium* oocysts g^-1^ of 40 (range 1–5,988) for adult cattle, 25 for juvenile cattle (range <1–17,467), 23 for adult sheep (range <1–152,474), 49 for juvenile sheep (range <1–641) and 54 for adult kangaroos (range <1–39,423) [84]. The age of the kangaroos and cattle sampled in the present study are unknown, but qPCR quantitation suggests that these were actual infections and not mechanical transmission. However, future studies should include oocyst purification via IMS prior to qPCR for more accurate quantitation. In addition, homogenisation of samples is important when comparing microscopy and qPCR i.e faecal slurries should be made, mixed well and aliquots of that mixture used for both microscopy and qPCR to ensure better consistency between techniques.

It is important to note that of the numbers of oocysts detected in animal faeces in catchments, only a fraction of oocysts may be infectious. For example, a recent study has shown that the infectivity fraction of oocysts within source water samples in South Australian catchments was low (~3.1%) [85]. While it would be expected that oocysts in faecal samples would have much higher infectivity than oocysts in source water, reports suggest that only 50% of oocysts in fresh faeces are infectious, and that temperature and desiccation can rapidly inactivate oocysts in faeces while solar inactivation, predation and temperature will all impact oocyst survival in water [86].

The identification of mostly zoonotic *Cryptosporidium* species in animals inhabiting Sydney catchments indicates that there is a need to diligently monitor *Cryptosporidium* in source waters. Such monitoring is also critical, given the resistance of *Cryptosporidium* oocysts to chlorine [87]. Further studies are essential to confirm the nature of the *C*. *hominis* variant detected in this study and to determine if it represents an infection risk for humans.

## Conclusions

Of the five *Cryptosporidium* species identified in this study, four species are of public health significance. The presence of zoonotic *Cryptosporidium* species in both livestock and wildlife inhabiting drinking water catchments may have implications for management of drinking water sources. Therefore, continued identification of the sources/carriers of human pathogenic strains would be useful to more accurately assess risk.
